# Study on corrosion behavior of X80 steel under stripping coating by sulfate reducing bacteria

**DOI:** 10.1186/s12896-020-00664-5

**Published:** 2021-01-09

**Authors:** Yan-Yu Cui, Yong-Xiang Qin, Qing-Miao Ding, Yu-Ning Gao

**Affiliations:** College of Airport, Civil Aviation University of China, Tianjin, 300300 P. R. China

**Keywords:** Sulfate reducing bacteria (SRB), Microbial corrosion, X80 steel, Electrochemistry

## Abstract

**Background:**

At present, microorganism has been considered as important factors that threaten to buried pipelines with disbonded coatings. Aiming at the problem of unknown corrosion mechanism of sulfate-reducing bacteria (SRB), a series of studies have been carried out in this paper. Spectrophotometer and fluorescent labeling technology are used to study the growth and attachment of SRB in the simulated soil solution. The corrosion behavior of X80 pipeline steel with or without SRB was researched by electrochemical methods such as open circuit potential, dynamic potential polarization curve, and electrochemical impedance spectroscopy. The microscopic morphology of the corrosion products on the surface was observed with a scanning electron microscope (SEM), and the element content of the corrosion products on the surface of the sample after corrosion was observed using X-ray energy spectrum (EDS) analysis.

**Results:**

The results showed that the growth and reproduction of SRB caused the pH of the soil simulated solution to increase, which may promote the corrosion of X80 steel. In addition, the cathode reaction of X80 steel in a sterile environment is the reduction of H^+^, and the main corrosion product is iron oxide. When the soil simulation solution contains SRB, the cathodic reaction is controlled by both H^+^ reduction and sulfide depolarization reactions, and FeS appears in the corrosion products.

**Conclusion:**

Although the life cycle of SRB is only about 14 days, the corrosion of X80 steel is greatly promoted by SRB, and even causes corrosion perforation, which will bring huge economic losses and serious safety hazards.

## Background

Microbiologically influenced corrosion (MIC) refers to microbial life activities that directly or indirectly promote the metal destruction caused by the corrosion process [1–5]. They can be found in various fields such as soil, machinery, oil fields and seawater. MIC is basically caused by bacteria, which is essentially electrochemical corrosion [6–12]. Generally, anti-corrosion coatings and cathodic protection are used in combination to deal with external corrosion of metal materials such as underground pipelines and oil storage tanks. This measure slowed the corrosion rate to a certain extent, but the corrosion problem still exists. In addition, if the anti-corrosion layer has defects such as shedding, the corrosion rate of the buried pipeline will be further accelerated [3, 13–15]. According to investigations, sulfate reducing bacteria, iron bacteria, iron oxidizing bacteria, and sulfur oxidizing bacteria have a great influence on metal corrosion, and SRB is considered to be one of the most influential microorganisms [16–19]. At present, national and international researchers have done many studies about SRB corrosion. Venzlaff et al. [20] used potential dynamics polarization measurement and found that the electrons released by the anode dissolution are directly transferred to the specific protein in the sulfate reduction site of the cell through the conductive ferrous sulfide to reduce the sulfate. This conclusion is consistent with the BCSR (Biocatalytic cathodic sulfate reduction) theory proposed by Enning [21]. Wikiel, Dong and Castaneda [19, 22, 23] believe that the multi-anisotropy of biofilm on the metal surface, the solution enters the bottom of the biofilm through the porous structure of the biofilm and directly contacts the metal, which makes the environment of the metal vary and local corrosion is more likely to occur. Brenda and Washizu [24, 25] believe that biofilm has a catalytic effect, which can increase the cathode current density and accelerate the self-passivation of metal surfaces.

However, due to the diversity of SRB and the complexity of its metabolism, the corrosion mechanism of SRB is still inconclusive. In addition, there are few reports on the effect of SRB corrosion on the metal substrate under the peeling coating. Therefore, it has practical engineering significance and application value to carry out the corrosion research of SRB in complex environment. Therefore, in order to provide a certain theoretical basis and data support for the safe operation of the pipeline, a series of technical meanswere used to study the influence of SRB on the corrosion behavior of X80 steel under peeling coating.

## Methods

### Sample preparation

The copper wire was welded to the back of the sample, and the side and welding surface were sealed with epoxy resin. The working surface was polished to the mirror surface with a series of emery paper (coarseness between 320 and 2000 mesh), and then distilled water, ethanol and acetone were used to wash it. The chemical composition (mass fraction) of X80 pipeline steel is shown in Table 1.

**Table 1:** Chemical composition of X80 steel (wt%)

C	Si	Mn	P	S	Cr	Ni	Ti	Nb	V	Mo	Fe
0.063	0.28	1.83	0.011	0.0006	0.03	0.03	0.016	0.061	0.059	0.22	For balance

### Experimental medium

The soil near the X80 pipeline being used in southwestern China was put into a drying box to air dry to remove debris, and then it was dried for 10 h to remove moisture in an oven at 120 °C. The dried soil and deionized water are mixed with a mass ratio of 1:5, and the titration test is performed by centrifugation [26]. The chemical composition and content of the soil are shown in Table 2. The soil simulation solution is prepared with deionized water, and nitrogen is filled into the solution to exhaust oxygen. Subsequently, it was placed in an autoclave (403 K) for about thirty minutes to kill the original microorganisms.

**Table 2:** Chemical composition of soil solution

H_2_O/mL	Na_2_CO_3_/g	NaCl/g	Na_2_SO_4_/g	NaHCO_3_/g
1000	0.16	0.5125	0.1712	0.0865

### Activation culture and inoculation of sulfate reducing bacteria

The Postgate medium (0.52 g), D-sodium lactate (0.11 g), and distilled water (100 mL) were measured and placed in a sterile Erlenmeyer flask, after which 5 mol/L NaOH was used to adjust the pH of the solution. The configured medium is sterilized in an autoclave, and then it is placed on an ultra-clean workbench to cool to room temperature, and the UV lamp is always turned on during the cooling process. Among them, the components of Postgate medium are divided into: Yeast extract 1.0 g/L, Sodium sulfate (Na_2_SO_4_) 3.5 g/L、Magnesium chloride hexahydrate (MgCl_2_·6H_2_O)2.0 g/L、Ascorbic acid(C_6_H_8_O_6_)0.1 g/L、Ammonium chloride (NH_4_Cl)1.0 g/L、Dipotassium phosphate(K_2_HPO_4_)0.5 g/L、Calcium chloride (CaCl_2_)0.05 g/L、Ferrous sulfate heptahydrate (FeSO_4_·7H_2_O)0.5 g/L、Sodium thioglycolate(C_2_H_3_NaO_2_S)0.1 g/L.

The purebred SRB was taken out of the refrigerator to thaw, and then 30 mL of pure bacterial solution (including glycerol) was removed with a pipette and added to the prepared liquid medium (100 mL) [27]. The inoculated medium was placed in a constant temperature biochemical incubator at 30 °C for cultivation, and the color change of the liquid medium was continuously observed. If it turns black completely, it indicates that the cultured SRB has biological activity. The experiment is divided into two groups: (a) the bacteria experimental group inoculated with SRB and (b) the sterile control group not inoculated with SRB. For the experimental group with bacteria, the cultured SRB solution (50 mL) and the simulated soil solution (950 mL) were mixed. For the sterile control group, deionized water 50 (mL) and simulated soil solution (950 mL) were mixed [26].

### Electrochemical experiment

**Fig. 1:**
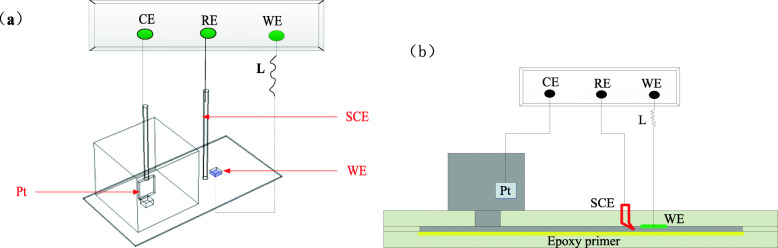
Schematic of experimental set-up: (**a**) Three-dimensional; (**b**) Two-dimensional

The epoxy primer was thermally sprayed onto the bottom plate of the experimental device to simulate the peeling of the coating. The schematic diagram of the experimental test device is shown in Fig. 1, where the peeling height of the coating is 1 mm. The electrochemical experiment uses a three-electrode test system, the electrochemical workstation model is CHI660D, in which X80 pipeline steel is used as the working electrode, the saturated calomel electrode is used as the reference electrode, and the Pt electrode is used as the auxiliary electrode. Electrochemical experiments mainly include open circuit potential (OCP), dynamic potential polarization curve, alternating current impedance (EIS) test [6]. First, the open circuit potential of the working electrode is tested, and then after it stabilizes, the electrokinetic polarization curve and EIS are tested. The scan rate of the polarization curve is 0.1 mV/s, the scan range is *Eocp* ± 250 mV, and the scan rate of EIS is 0.5 mV/s, the test frequency range is 10^− 2^ ~ 10^6^, and the AC excitation signal is a 10 mV sine wave.

### Soak experiment

The prepared electrode was immersed in the experimental device for 10 days, and then it was fixed with 2% glutaraldehyde phosphate buffer for about 5 h. After the fixation is completed, rinse with sterile saline and use alcohol of 25, 50, 75, and 100% volume fraction to dehydrate about 10 min [27]. After dehydration, use the model EMS 850 critical point dryer for drying. After dehydration was completed, we used the model EMS 850 critical point dryer for drying. Then use the environmental field SEM of model KYKY-EM6X00 to observe the micro-morphology of the corrosion products on the surface of the sample, and EDS to analysis the elemental of the corrosion products on the surface. The applied SEM and EDS working parameters are as follows: the imaging method was secondary electron imaging (SEI), acceleration voltage was 15,000 V, working distance was 7500 μm and the emission current was 159 μA. Furthermore, the EDS system was energy dispersive type and the beam current was 40 nA.

### Weightlessness experiment

First, the sample was weighed with a high-precision electronic analytical balance model FA2005N. After the sample was soaked, it was rinsed with deionized water and immersed in a rust removal solution (500 mL concentrated hydrochloric acid + 500 mL distilled water + 3.5 g hexamethylenetetramine) at room temperature for 10 min to remove surface corrosion product. After the rust removal is complete, the sample is cleaned with deionized water and absolute ethanol, and then completely dried (the difference between the two measurements is within 0.0002 g can be considered sufficiently dry), and finally weighed again. The corrosion rate is calculated.

## Result

### Growth of SRB

**Fig. 2:**
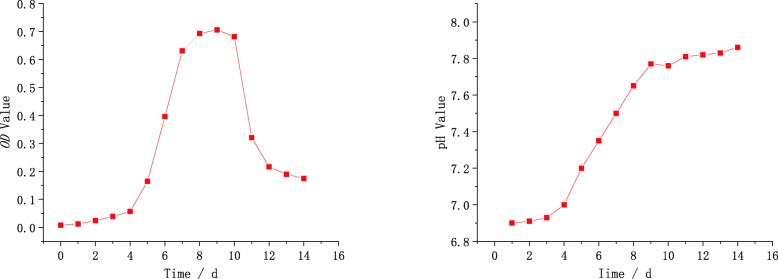
Variations of *OD* value and pH value with time: (**a**) *OD* value; (**b**) pH value

An 754PC ultraviolet spectrophotometer was used to measure the optical density (*OD*) value of the solution after X80 was immersed in the soil for different days [28], the measurement results are shown in Fig. 2. It can be seen from Fig. 2 (a) that SRB are the adaptation period from the first day to the forth. This stage shows that SRB do not rapidly multiply and die after entering the new environment (soil simulation solution) from the culture medium and its number is relatively stable overall. From the forth days to the eighth, SRB enter logarithmic growth phase, and the growth rate of SRB are the fastest in this phase. From the eighth days to the tenth, SRB enter stable growth period, and the number of SRB reached the maximum. From the thirteenth days to the fourteenth, SRB enters the residual stage, in which the number of SRB is relatively stable, and the total number remains at a low level. In addition, hydrogen ions are continuously consumed during the growth and reproduction of SRB, which leads to a continuous increase in the pH of the solution. This conclusion is consistent with the experimental results in Fig. 2 (b).

**Fig. 3:**
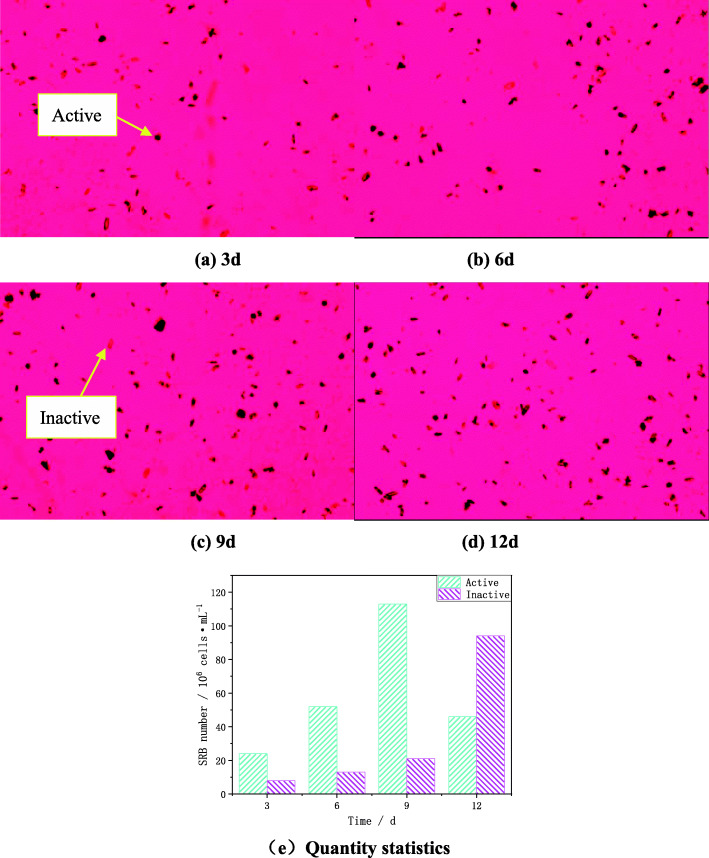
The number of SRB on the surface changes with the immersion time: (**a**) 3d; (**b**)6d; (**c**) 9d; (**d**) 12d; (**e**) Quantity statistics

In addition, acridine orange was used to fluorescently label the SRB on the sample surface and it can specifically bind to nucleic acid substances in cells after contact with bacteria. At the same time, Imagepro Plus software was used to count the number of SRB attached to the sample surface and the results are shown in Fig. 3 [26] (the magnification is 500 times). It can be seen from Fig. 3 that the amount of SRB attached to the surface of the sample increases with the increase of the soaking time. At the third day, the amount of SRB attached to the surface of the sample was small and partially inactivated. At the sixth day, the amount of SRB adhered to the surface of the sample increased, and the number in the inactivated state decreased. At the ninth day, the amount of SRB adhesion on the surface of the sample significantly increased. At the twelfth day, the amount of SRB adhesion on the surface of the sample continued to increase, but most of them are in an inactive state.

### Effect of SRB on the electrochemical behavior of X80 steel

**Fig. 4:**
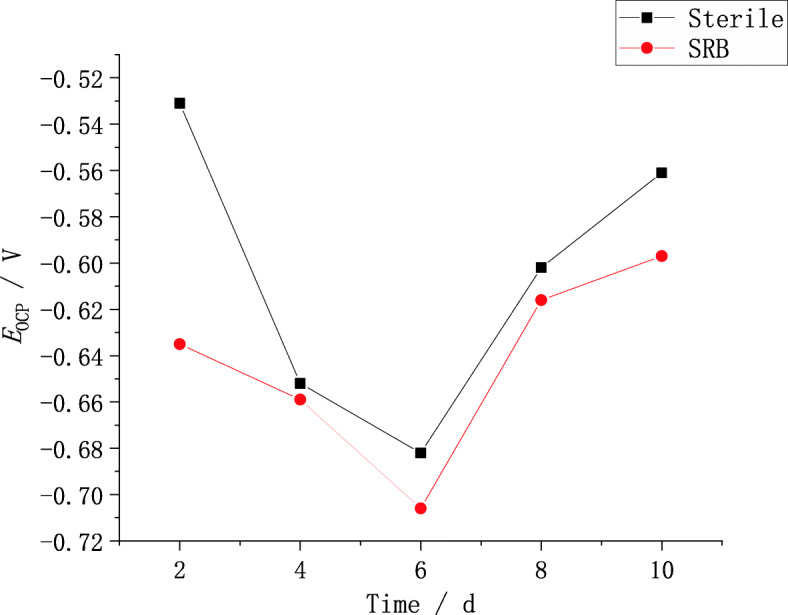
Open circuit potential of X80 steel in bacteria and sterile environment

The open circuit potential (*E*_*OCP*_) of X80 pipeline steel in bacteria and sterile soil simulation solution over time is shown in Fig. 4. It can be found from Fig. 4 that the open circuit potential of X80 steel changes with time in the same way in the soil solution containing SRB as in the sterile solution. As the immersion time increases, the open circuit potential first moves negatively and then positively [12]. Meanwhile, under the same immersion time, the open circuit potential in the SRB-containing solution is always negative than that in the sterile solution. In other words, the corrosion tendency of X80 steel is increased due to the presence of SRB, and the corrosion tendency reaches the maximum around the 6th day.

**Fig. 5:**
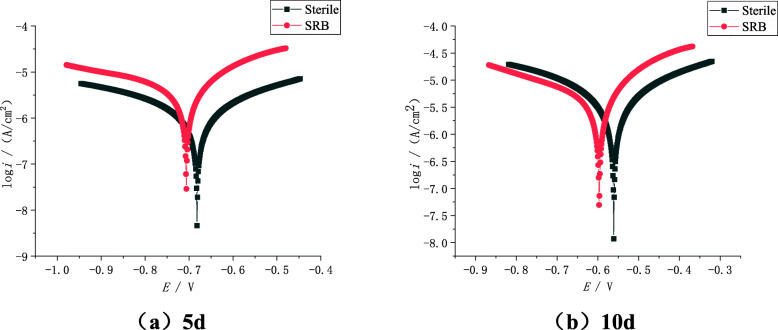
Potential polarization curve of X80 steel in sterile and bacteria soil simulation solution

The polarization curve of X80 steel after immersion for 5 days and 10 days are shown in Fig. 5. It can be seen from Fig. 5 that the polarization curve of X80 steel in the solution containing SRB is to the upper left, which indicates that X80 steel has a greater corrosion tendency. The polarization curve was fitted and the corresponding electrochemical parameters are listed in Table 3. It can be seen from Fig. 5 and Table 3 that the corrosion current density of X80 steel in the solution containing SRB is about 7 times that of the sterile environment during the same immersion time, which indicates that SRB greatly promotes corrosion. In addition, the results of the experimental group and the control group showed that the corrosion rate of X80 steel on the 5th day was lower than that on the 10th day.

**Table 3:** Fitting results of polarization curves in sterile and bacteria soil simulated solutions

Environment	Time (d)	*E*_corr_ (V)	*i*_corr_ (μA/cm^2^)	***β***_a_ (mV·dec^−1^)	***β***_c_ (mV·dec^−1^)	***β***_a_/***β***_c_
Sterile	5	−0.682	1.064	727.0	651.4	1.12
	10	−0.602	1.535	711.9	669.4	1.06
SRB	5	−0.647	7.922	735.4	659.1	1.16
	10	−0.552	10.95	719.5	663.1	1.09

**Fig. 6:**
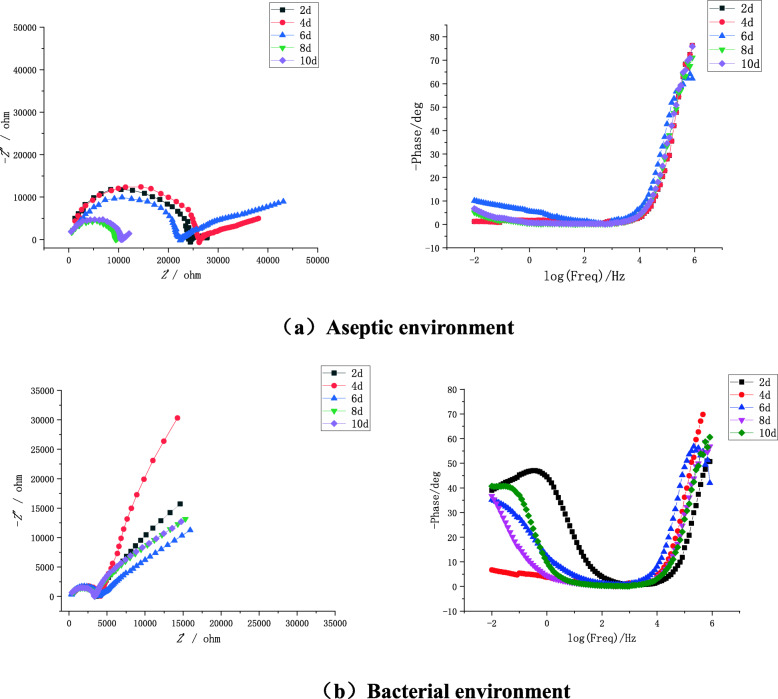
Nyquist diagram and Bode diagram of X80 steel in a sterile and SRB-containing environment. **a** Aseptic environment. **b** Bacterial environment

In order to further research the effect of SRB on the corrosion behavior of X80 steel in the soil simulation solution, the AC impedance spectrum of X80 steel was measured and the results are shown in Fig. 6. Where Z′ is the real part of the AC impedance spectrum and Z″ is the imaginary part [29]. It can be seen from Fig. 6(a) that with the extension of the sample immersion time, both the impedance arc and the capacitive reactance radius of X80 steel show a law that first slightly increases and then gradually decreases. This is because the corrosion product continues to increase over time from the first day to the fourth and its diffusion rate is much lower than the generation, which leads to the accumulation of corrosion product film on the surface of X80 steel, and the corrosion product plays a role in protecting the substrate. From the fourth day to the tenth, as the diffusion rate of the corrosion product increases and the generation rate decreases, the corrosion product film on the metal surface begins to crack or even fall off. This causes harmful ions in the solution to contact the remaining X80 substrate through the gap to form crevice corrosion and accelerate the corrosion rate of the substrate. On the other hand, the combination of Fe^2+^ and OH^−^ in the solution forms Fe (OH)_2_, which will form galvanic corrosion with X80 steel to further accelerate the corrosion rate. As SRB continues to increase from the fourth day to the eighth, the impedance arc and capacitive reactance radius of X80 steel gradually decrease, which is a manifestation of the increase in the corrosion rate of the sample caused by the life activity of SRB [30]. From the eighth day to the tenth, SRB formed a dense biofilm on the surface of X80 steel, which caused the corrosion rate of the substrate to decrease. At the same time, the sulfide produced by SRB during growth and metabolism attaches to the gap of the biofilm, which further strengthens the effect of the biofilm on the surface of X80 steel on the diffusion of solution ions [31]. In addition, the presence of straight line is warburg impedance in the high frequency region, which This shows that there is a diffusion control process in the electrochemical corrosion process. According to Fig. 6, it can be found that the impedance radius is significantly smaller in the SRB-containing environment under the same immersion time, which once again proves that X80 steel is more prone to corrosion in the soil containing SRB.

**Fig. 7:**
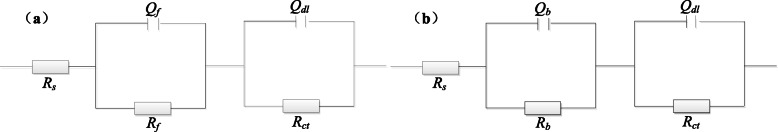
The equivalent circuits of X80 steel: (**a**) sterile soil; (**b**) Soil inoculated with SRB

**Fig. 8:**
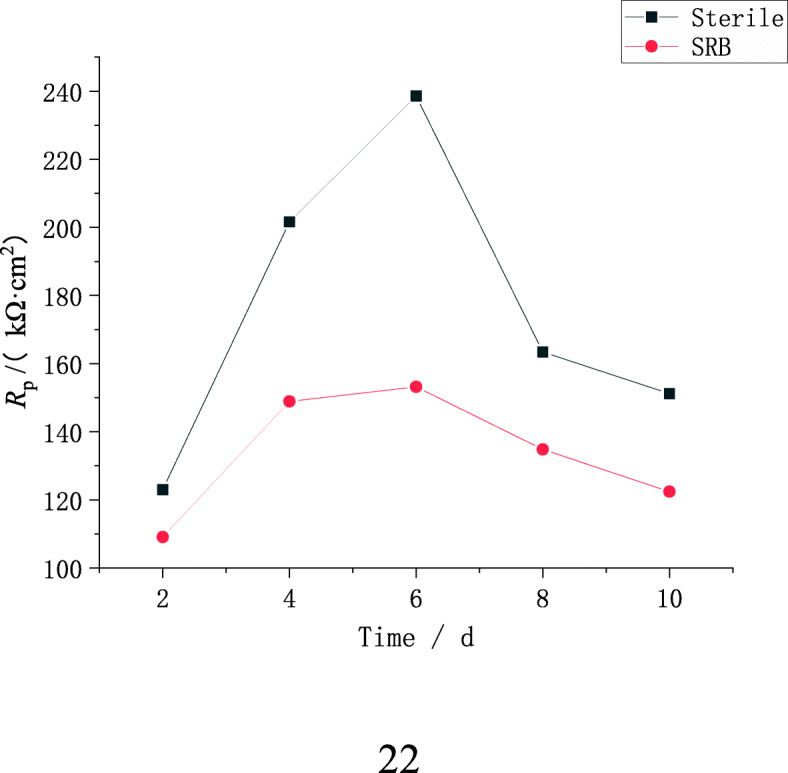
Polarization resistance changes with time of X80 steel in inoculated and uninoculated SRB soil

In order to analyze the impedance spectrum in a more intuitive way, ZSimWin software is used to fit the impedance spectrum, where the equivalent circuit is shown in Fig. 7, and the fitting result is shown in Tables 4 and 5 and Fig. 8. Where *R*_*s*_ is the resistance of the simulated soil solution, *R*_*b*_ and *R*_f_ represent the resistance of the corrosion products, *R*_*ct*_ represents the charge transfer resistance, *Q*_*dl*_ represents the electric double layer capacitance, *Q*_*b*_ and *Q*_*f*_ represent different meanings, *Q*_*b*_ represents the corrosion product film formed capacitive reactance in the sterile soil. While *Q*_*f*_ is the capacitive resistance formed under the combined action of microbial film and corrosion product film in the soil of containing SRB. The influence of state variable electrode potential and corrosion product layer or biofilm is considered, so the polarization resistance *R*_*p*_ *=* (*Z*_*F*_)_w = 0_ *= R*_f_ *+ R*_c*t*_ is used to reflect the corrosion rate of X80 steel in the system [32]. Where *Z*_*F*_ is the Faraday impedance of the experimental system, which is equivalent to the impedance of the experimental system after removing the soil simulated solution resistance *R*_s_ and the non-Faraday impedance *Z*_*NF*_.

**Table 4:** Fitting results of EIS in sterile soil simulated solutions

Time(d)	R_s_ (Ω·cm^2^)	n_f_	R_f_ (kΩ·cm^2^)	n_dl_	R_ct_ (kΩ·cm^2^)
5	626.0	0.28	12.4	0.9287	120.8
10	460.2	0.67	10.3	0.4337	112.1

**Table 5:** Fitting results of EIS in simulated soil solution containing bacteria

Time(d)	R_s_ (Ω·cm^2^)	n_f_	R_b_ (kΩ·cm^2^)	n_dl_	R_ct_ (kΩ·cm^2^)
5	285.0	0.96	3.59	0.531	135.0
10	164.8	0.71	1.48	0.981	2.999

It can be seen from Tables 4 and 5 that the presence of SRB will significantly reduce the soil resistance, that is to say, the electrolyte conductivity will increase. On the other hand, *R*_f_ is always greater than *R*_b_ during the same immersion time, which also shows that the presence of SRB reduces the corrosion resistance of X80 steel in soil. It can be seen from Fig. 8 that the polarization resistance *R*_*p*_ of X80 steel in aseptic and SRB-containing soils changes in the same way, which are both increase first and then decrease as the immersion time increases, and the maximum is at the 6th day. This is because the formation rate of corrosion products in the early stage of the experiment is much greater than its diffusion rate, which leads to the gradual accumulation of corrosives on the sample surface, and the formed corrosion product film inhibits the corrosion of metals. As the immersion time continues to increase, the corrosion product diffusion rate exceeds its generation rate, and defects such as cracks appear on the corrosion product film, which in turn leads to a decrease in polarization resistance. In addition, SRB also promotes the diffusion of corrosion products, which leads to a smaller *R*_*p*_ of the sample in the soil containing SRB. This conclusion indicates that SRB promotes the corrosion of X80 steel, which is consistent with the analysis results of open circuit potential and polarization curve.

### Corrosion product morphology and product analysis

**Fig. 9:**
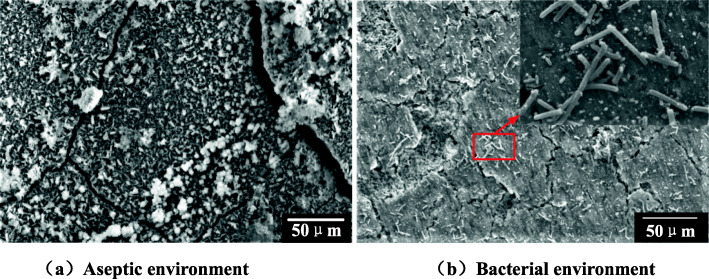
Micro-morphology of corrosion products on the sample surface. Acceleration voltage was 15,000 V, working distance was 7500 μm, emission current was 159 μA. **a** Aseptic soil environment. **b** Bacterial soil environment

**Fig. 10:**
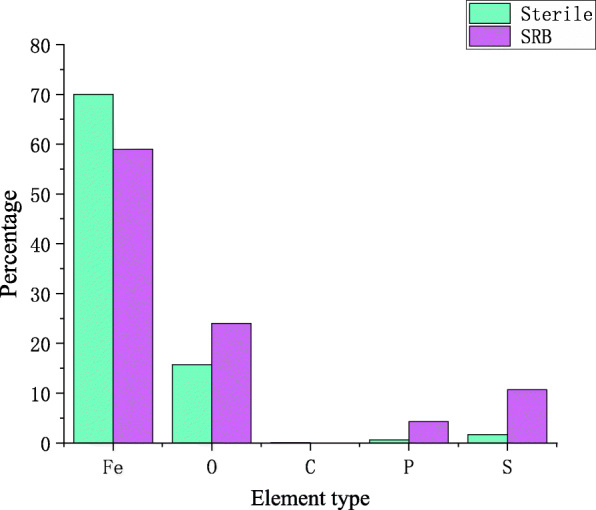
EDS analysis results of corrosion products in inoculated and uninoculated SRB soil

The microscopic morphology of the corrosion products of X80 steel after immersion for ten days is shown in Fig. 9. The area of Fig. 9 was scanned by EDS, and the result is shown in Fig. 10.

**Fig. 11:**
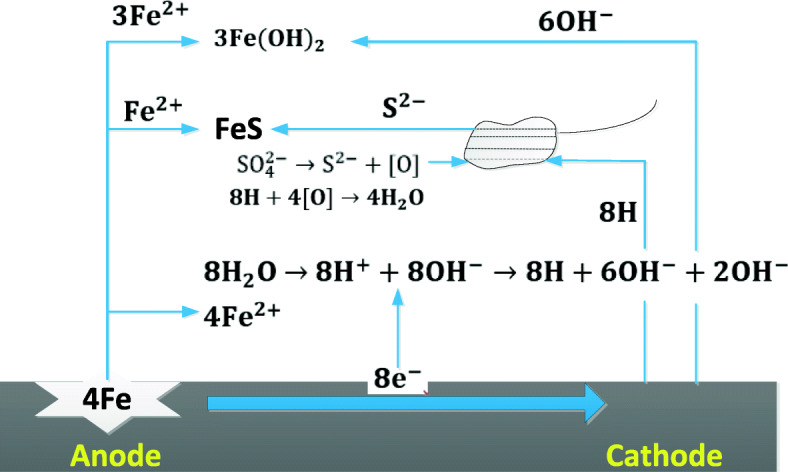
SRB corrosion diagram

It can be seen from Fig. 9 that after X80 steel is placed in sterile soil for ten days, a small part of the “cotton ball”-like product α-FeO (OH) and the typical “flower-like” scale γ-FeO (OH) morphology on the sample surface. In the soil of containing SRB, a large amount of SRB on the surface of the sample was clearly observed, and a dense corrosion product film was formed. It can be seen from Fig. 10 that when SRB is present in the soil, the proportion of iron on the surface of the sample decreases, and the proportion of oxygen, phosphorus and sulfur increases significantly [33]. This is because the corrosion products of X80 steel in sterile soil are mainly oxidation Iron, while SRB is present in the soil, sulfide of iron and phosphide of iron will be formed on the surface of the sample [7]. Therefore, the cathodic reaction process of the corrosion of X80 steel in SRB-containing soil is the depolarization reaction of phosphide and sulfide, which further proves that SRB promotes the corrosion of X80 steel [34]. The corrosion mechanism are shown in eqs. (1) to (6), and the specific reaction path is shown in Fig. 11 [3].

1 $$ \mathrm{Anode}\ \mathrm{reaction}\kern1.75em 4\mathrm{Fe}\to 4{\mathrm{Fe}}^{2+}+8{\mathrm{e}}^{-} $$

2 $$ \mathrm{Ionization}\ \mathrm{of}\ \mathrm{water}\kern1em 8{\mathrm{H}}_2\mathrm{O}\leftrightarrow 8{\mathrm{H}}^{+}+8\mathrm{O}{\mathrm{H}}^{-} $$

3 $$ \mathrm{Cathodic}\ \mathrm{reaction}\kern1.25em 8{\mathrm{H}}^{+}+8{\mathrm{e}}^{-}\to 8\mathrm{H} $$

4 $$ \mathrm{Cathode}\ \mathrm{reaction}\ \mathrm{with}\ \mathrm{bacteria}\kern1.25em \mathrm{S}{\mathrm{O}}_4^{2-}+8\mathrm{H}\ \overset{\mathrm{S}\mathrm{RB}}{\to }\ {\mathrm{S}}^{2-}+4{\mathrm{H}}_2\mathrm{O} $$

5 $$ \mathrm{Primary}\ \mathrm{corrosion}\ \mathrm{products}\kern1.25em \mathrm{F}{\mathrm{e}}^{2+}+{\mathrm{S}}^{2-}\to \mathrm{FeS} $$

6 $$ \mathrm{Secondary}\ \mathrm{corrosion}\ \mathrm{products}\kern0.75em 3\mathrm{F}{\mathrm{e}}^{2+}+6\mathrm{O}{\mathrm{H}}^{-}\to 3\mathrm{F}\mathrm{e}{\left(\mathrm{OH}\right)}_2 $$

### Corrosion weight gain

**Fig. 12:**
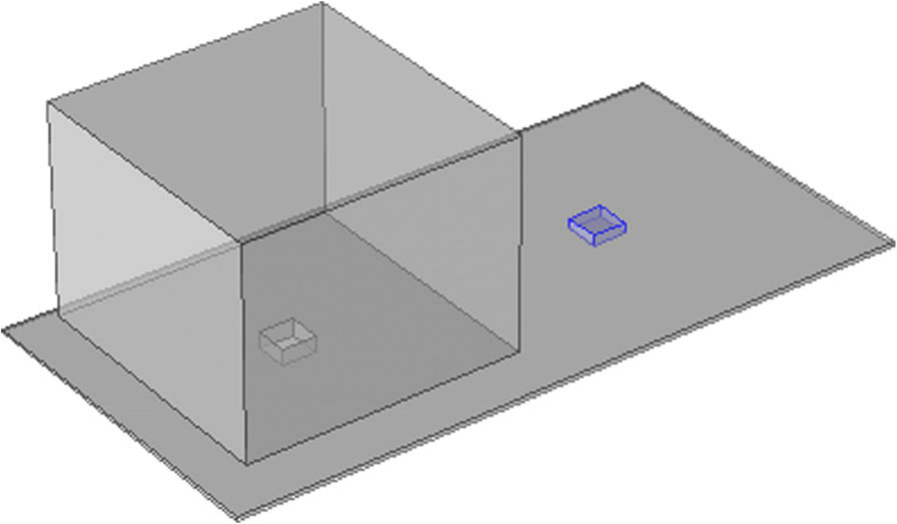
Corrosion rate of X80 pipeline steel with or without SRB in the soil

The corrosion rate of X80 pipeline steel under the peeling coating in the aseptic and SRB-containing soil was measured and calculated by the weightlessness experiment method. The results are expressed in terms of mass change as shown in Fig. 12. It can be seen from Fig. 12 that the corrosion rate of X80 steel in SRB-containing soil is greater than that of aseptic soil in the same time, which indicates that SRB greatly promotes corrosion. In addition, the results of the experimental group and the control group once again showed that the corrosion rate of X80 steel on the 5th day was lower than that on the 10th day.

### Numerical simulation

**Fig. 13:**
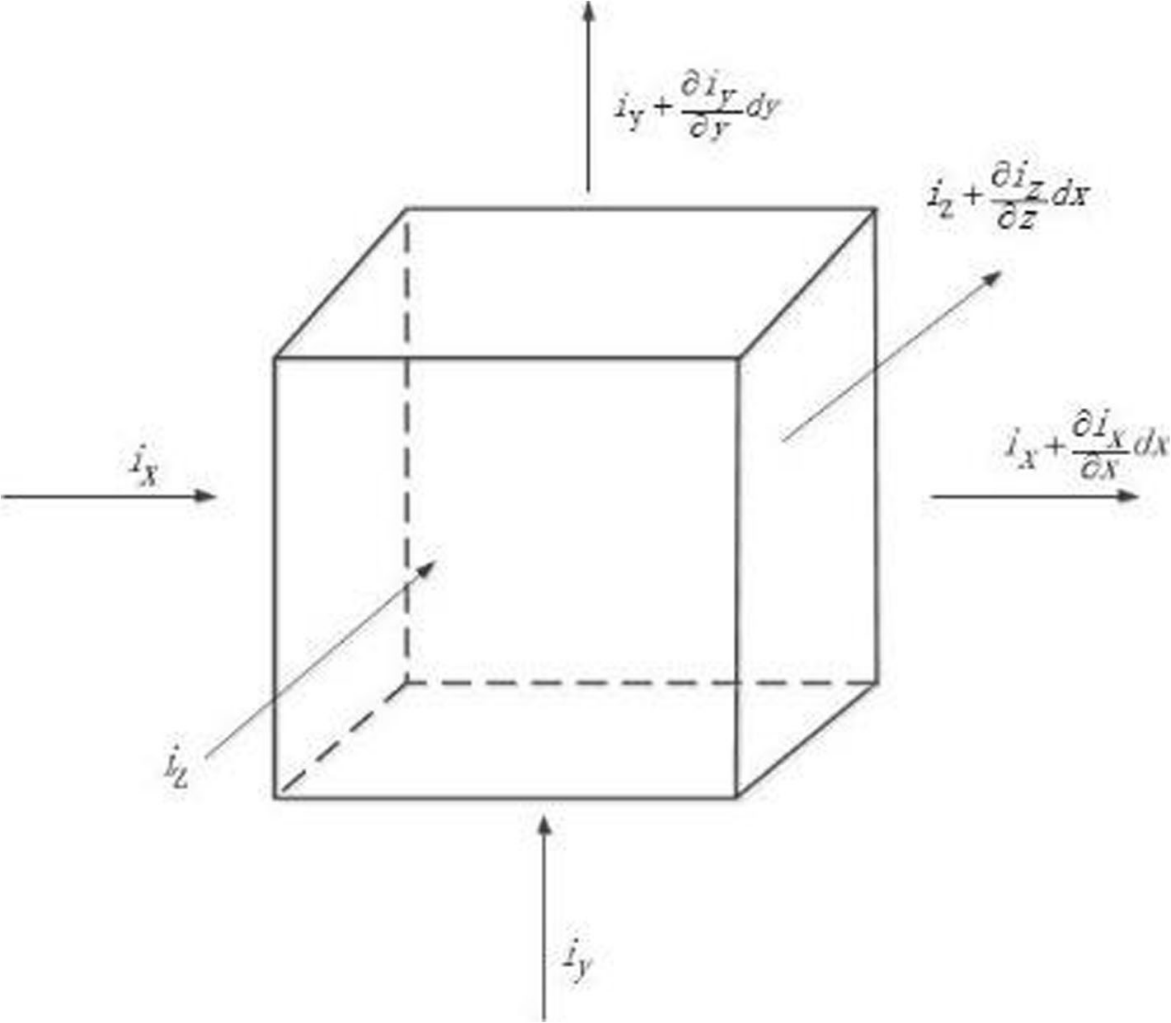
Schematic diagram of micro-body current flow

The “secondary current distribution and dilute matter transfer” interface were used to simulation and the three-dimensional physical model was established as shown in Fig. 12. Arbitrarily take a microcell from the stable, passive corrosion model shown in Fig. 12, its side length are set as *d*_x_, *d*_y_, *d*_z_, where the volume *d*_v_ = *d*_x_*d*_y_*d*_z_ ≠ 0, and the microcell structure is shown in Fig. 13.

Suppose that the current flowing into the cell in the *x* direction and the current flowing out in the model are equal, namely:

7 $$ {i}_x dydz=\left({i}_x+\frac{\partial {i}_x}{\partial x} dx\right) dydz $$

Similarly, in the *y* direction:

8 $$ {i}_y dxdz=\left({i}_y+\frac{\partial {i}_y}{\partial y} dy\right) dxdz $$

in the *z* direction:

9 $$ {i}_z dxdy=\left({i}_z+\frac{\partial {i}_z}{\partial z} dz\right) dxdy $$

From the above eqs. (7), (8), (9) we can get:

10 $$ -\frac{1}{\rho }{\nabla}^2\varphi =0 $$

**Fig. 14:**
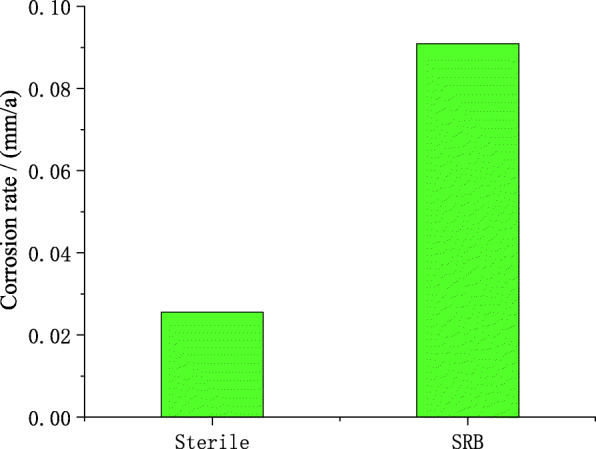
Corrosion rate of X80 steel

That is to say, the Laplace equation is used as the control equation of the potential distribution in the corrosion field. The surface corrosion rate of X80 steel obtained by simulation calculation after 10 days in soil is shown in Fig. 14. It can be found from Fig. 14 that the maximum corrosion rate of X80 steel in soil containing SRB is about 3.5 times that of sterile soil.

## Conclusions

1. The growth and reproduction of SRB will cause the pH of the solution to rise. The open circuit potential change law of X80 steel in aseptic and SRB-containing soil is basically the same, but the corrosion tendency is greater in SRB-containing soil.
2. Both the simulation results and the electrochemical experiment results show that SRB promotes the corrosion of X80 steel in the soil. In addition, the simulation results show that the maximum corrosion rate with SRB is about 3.5 times that of sterile soil.
3. The corrosion products of X80 steel in sterile soil are mainly oxidation of iron, while SRB is present in the soil, sulfide and phosphide of iron will be formed on the surface of the sample

## Data Availability

All data, raw and processed, is readily available from the corresponding author on request.

## Abbreviations

SRB: Sulfate-reducing bacteria
SEM: Scanning electron microscope
EDS: X-ray energy spectrum
MIC: Microbiologically influenced corrosion
BCSR: Biocatalytic cathodic sulfate reduction
